# Monocyte‐to‐Albumin Ratio (MAR) Is Associated With All‐Cause Mortality in Adult Stroke Patients: Evidence From NHANES (1999–2018)

**DOI:** 10.1155/srat/5461096

**Published:** 2026-08-25

**Authors:** Jia Wang, Yue Wu, Jing Xia

**Affiliations:** ^1^ School of Public Health, Wuhan University of Science and Technology, Wuhan, China, wust.edu.cn; ^2^ Xuanhan County People′s Hospital, Dazhou, Sichuan, China; ^3^ People′s Hospital of Xiangxi Tujia and Miao Autonomous Prefecture, Jishou, Hunan Province, China; ^4^ Hubei Provincial Center for Disease Control and Prevention, Wuhan, China, cdc.gov

**Keywords:** all-cause mortality, monocyte-to-albumin ratio, NHANES, stroke

## Abstract

**Objective:**

To investigate the association between the monocyte‐to‐albumin ratio (MAR) and all‐cause mortality risk in stroke patients.

**Methods:**

We analyzed data from 2022 stroke participants (aged ≥ 20 years) in the National Health and Nutrition Examination Survey (NHANES) 1999–2018. MAR was calculated as the monocyte count (×10^9^/L) divided by serum albumin (g/L). The outcome was all‐cause mortality of stroke population. Multivariable Cox proportional hazards models were used to examine the association between MAR and all‐cause mortality, with restricted cubic splines assessing dose–response relationships and Kaplan–Meier curves with log‐rank tests evaluating cumulative survival probabilities. Furthermore, we compared the incremental predictive value of MAR against a conventional risk model (incorporating demographics and comorbidities) using the area under the curve (AUC), integrated discrimination improvement (IDI), and net reclassification improvement (NRI). Subgroup analyses and sensitivity analyses were performed, stratified by demographic and clinical characteristics, to verify robustness.

**Results:**

A total of 2022 participants were included, of whom 1016 (50.2%) were male and 1506 (74.5%) were aged ≥ 60 years. In multivariable Cox regression adjusting for demographic, lifestyle, and clinical covariates, elevated MAR was independently associated with all‐cause mortality (per 1‐unit increase: HR = 1.35, 95% CI: 1.21–1.50; T3 vs. T1: HR = 1.32, 95% CI: 1.12–1.55; both *p* < 0.05). Restricted cubic spline analysis revealed a nonlinear dose–response relationship (*p* for nonlinearity = 0.001). Kaplan–Meier curves demonstrated lower survival probabilities in the highest MAR tertile (T3) compared to the lowest (T1) (log‐rank *p* < 0.001). With respect to model performance, incorporating MAR into the conventional risk model resulted in a statistically significant but modest improvement in discrimination (*Δ* *A* *U* *C* = 0.0158) and reclassification accuracy (IDI = 0.0129, NRI = 0.208; all *p* < 0.05). Subgroup and sensitivity analyses confirmed the robustness of this association across various strata.

**Conclusion:**

Elevated MAR is independently associated with long‐term mortality in stroke survivors, reflecting underlying inflammation and nutritional insufficiency. These findings support MAR′s potential utility as a research tool in prospective cohort studies.

## 1. Introduction

Stroke remains a leading cause of long‐term disability and mortality worldwide, imposing a substantial global health burden [1, 2]. While traditional vascular risk factors are well‐established [3], emerging evidence highlights the pivotal role of systemic inflammation and nutritional decline in driving poststroke adverse outcomes [4, 5]. This evolving understanding has spurred a paradigm shift toward identifying cost‐effective, integrative biomarkers that capture the pathophysiological interplay between metabolic perturbations and inflammatory responses.

The monocyte‐to‐albumin ratio (MAR), calculated as the ratio of absolute monocyte count to serum albumin concentration, has emerged as a promising composite index. Unlike isolated markers, MAR simultaneously reflects pro‐inflammatory burden and nutritional reserve. Its clinical utility has been substantiated across diverse fields: In oncology, it aids in distinguishing malignant from benign conditions [6]; in cardiovascular medicine, it predicts all‐cause mortality in the general population [7]; and in acute cerebrovascular events, it correlates with short‐term functional outcomes [8, 9]. Biologically, MAR encapsulates a vicious cycle where pro‐inflammatory cytokines (e.g., TNF − *α* and IL − 1*β*) suppress albumin synthesis, whereas hypoalbuminemia diminishes antioxidant defenses, collectively exacerbating endothelial dysfunction [10–12].

Despite these advances, evidence specifically addressing MAR in stroke survivorship remains limited. Most published studies have focused on short‐term functional recovery or in‐hospital outcomes, often constrained by single‐center designs and small sample sizes [13, 14]. Notably, while several recent NHANES‐based investigations have examined related inflammation‐ or nutrition‐inflammation–based indices—such as NLR, NPAR, and Naples Prognostic Score—these studies differ from ours in key aspects, including the specific biomarker (exposure) and the long‐term all‐cause mortality endpoint [14–16]. Consequently, data directly linking MAR to long‐term survival specifically among stroke survivors remain scarce (Table S1).

To fill this critical gap, we leveraged the NHANES‐linked National Death Index (NDI) to examine the association between baseline MAR and long‐term all‐cause mortality in a nationally representative cohort of US stroke survivors—a population distinct from those examined in prior MAR or broader NHANES‐based biomarker studies.

## 2. Materials and Methods

### 2.1. Data Collection, Participants, and Variables

**Figure 1:**
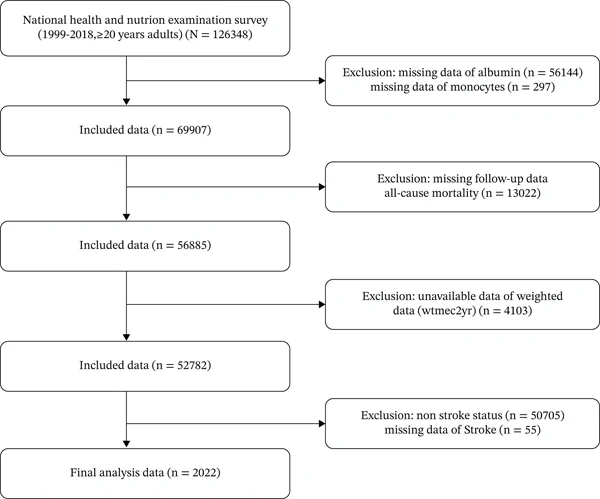
Flowchart of the participant′s selection from NHANES 1999–2018.

This study utilized data from the National Health and Nutrition Examination Survey (NHANES) cycles from 1999 to 2018. NHANES employs a multistage, stratified, complex sampling design, and the study protocol was approved by the National Center for Health Statistics (NCHS) Ethics Review Board, with all participants providing informed consent [17]. Detailed information regarding databases, laboratory procedures, and quality control measures has been reported in previous literature [18, 19]. From an initial 126,348 participants, we excluded individuals with missing data on monocyte count or albumin (*n* = 56,441), those without mortality linkage data (*n* = 13,022), those lacking weight data (*n* = 4103), and those without a self‐reported stroke history (*n* = 50,760). Ultimately, 2022 adults with stroke were included in the final analysis (Figure 1). Stroke status was determined based on self‐reported physician diagnosis during structured face‐to‐face interviews. Participants were classified as having a stroke if they answered “yes” to the question: “Has a doctor or other health professional ever told you that you had a stroke?” [20]. The primary exposure was the MAR, calculated at baseline as MAR = monocyte count (×10^9^/L)/serum albumin (g/L) [21]. The primary outcome was all‐cause mortality, which refers to the total number of deaths from any cause that occur within a specified time frame, determined via linkage to the NDI. Survival time was calculated from the date of the NHANES interview to the date of death or the end of follow‐up (December 31, 2019).

### 2.2. Covariates

Covariates were selected through a three‐pronged approach: clinical relevance, prior literature, and statistical multicollinearity assessment (variance inflation factor, VIF) to ensure robustness and parsimony in the regression models (Table S1). The demographic data include variables such as age, gender, race, education level, and marital status. Participants were divided into two age categories: those under 65 years old and those 65 years or older [22] Marital status was categorized as either married/cohabiting or single/divorced/widowed [23]. Additional covariates included the poverty‐income ratio (PIR), body mass index (BMI, measured in kg/m^2^) [24], smoking status (never smoked, former smoker, or current smoker) [25], drinking status (nondrinker, former drinker, or current drinker) [26], and medical history (encompassing conditions such as hypertension, congestive heart disease, congestive heart failure, hyperlipidemia, and diabetes) [27]. Based on the VIF screening results, blood urea nitrogen (BUN, mg/dL), serum creatinine (Cr, mg/dL), total cholesterol (TC, mmol/L), and uric acid (UA, *μ*mol/L) were selected for this analysis. All laboratory measurements were performed at the NHANES Mobile Examination Center by trained personnel following standardized protocols. Detailed laboratory procedures are published in the “NHANES Laboratory/Medical Technologists Procedures Manual,” whereas specific descriptions of the laboratory assay protocols and covariate definitions are provided in Text S1. These variables were analyzed as continuous measures in all regression models.

### 2.3. Statistical Analysis

In accordance with NHANES analytical guidelines, descriptive statistics in Table 1 were weighted using the appropriate interview/examination weights (WTINT2YR/WTMEC2YR) and accounted for clustering (SDMVPSU) and stratification (SDMVSTRA) to generate nationally representative estimates. Sampling weights were recalculated for the combined 1999–2018 cycles as previously described (2/10 × WTMEC4YR for 1999–2002; 1/10 × WTMEC2YR thereafter). Although weighting is essential for prevalence estimation, it can substantially increase variance and attenuate effect estimates in multivariable regression—particularly in outcome‐rare or subgroup analyses—and may introduce overadjustment when covariates are strongly correlated with sampling probabilities [10, 28]. Because our primary objective was to evaluate the internal association between MAR and mortality rather than to derive population‐level prevalence, and because key demographic variables correlated with survey weights (age, sex, and race/ethnicity) were rigorously adjusted for in all models, we present unweighted Cox regression and restricted cubic spline (RCS) analyses as the main results to preserve statistical power and estimate stability, consistent with previous NHANES‐based survival analyses [29, 30]. Survey‐weighted models are provided as sensitivity analyses in Table S3.

**Table 1:** Characteristics of the study patients according to monocyte‐to‐albumin ratios (MAR).

Variables	MAR				
	Total, *N* = 2022(100%)^a^	T1 (< 1.19) *n* = 649(30.2%)^a^	T2 (1.19–1.62) *n* = 696(33%)^a^	T3 (≥ 1.62) *n* = 677(36.8%)^a^	*p* ^*b*^
Weighted data, no	11,430,170.5	3,435,932.7	3,786,610.8	4,207,627.0	
Age years, mean ± SD	65.01 (14.90)	62.02 (15.40)	64.36 (14.05)	68.04 (14.67)	0.009
Sex, %					0.129
Male	1016 (43.0)	297 (37.3)	335 (42.7)	384 (47.9)	
Female	1006 (57.0)	352 (62.9)	361 (57.3)	293 (52.1)	
Race, %					0.123
Mexican American	1048 (73.2)	281 (66.4)	368 (76.1)	399 (76.3)	
Non‐Hispanic Black	493 (12.8)	197 (16.1)	163 (11.8)	133 (11.1)	
Non‐Hispanic White	252 (3.9)	93 (4.1)	93 (4.7)	66 (3.1)	
Other Hispanic	109 (4.5)	39 (8.1)	41 (2.5)	29 (3.4)	
Other	120 (5.6)	39 (5.6)	31 (4.9)	50 (6.1)	
Married status, %					
Married/living with partner	1046 (56)	327 (61.1)	367 (57.1)	352 (50.9)	
Divorced/single/living alone	938 (44)	306 (38.9)	317 (42.9)	315 (49.1)	
PIR, median (IQR)	2.36 (1.44)	2.47 (1.55)	2.48 (1.43)	2.16 (1.33)	
Education (years), %					
< 9	380 (13.0)	120 (12.0)	138 (12.1)	122 (14.6)	
9–11	398 (19.8)	131 (21.1)	130 (21.0)	137 (17.7)	
High school graduate	512 (29.8)	151 (26.7)	180 (30.1)	181 (31.9)	
College	490 (24.7)	163 (26.3)	168 (23.9)	159 (24.0)	
College graduate or above	239 (12.8)	82 (13.9)	80 (12.9)	77 (11.8)	
Smoke status, %					
Never	833 (43.9)	306 (48.3)	284 (42.9)	243 (41.1)	
Former	742 (32.7	225 (32.3)	243 (29.9)	274 (35.5)	
Current	445 (23.4)	117 (19.4)	169 (27.1)	159 (23.4)	
Drink status, %					
Never	311 (21.1)	103 (19.7)	116 (22.6)	92 (22.6)	
Former	654 (32.5)	205 (25.5)	213 (31.9)	236 (38.8)	
Current	825 (45.7)	283 (54,8)	281 (45.5)	261 (38.6)	
BMI kg/m^2^, Mean ± SD	29.72 (6.37)	29.30 (6.26)	29.73 (6.34)	30.07 (6.48)	
Angina (yes)	259 (17.6)	78 (15.9)	89 (18.7)	92 (18.0)	
Heart attack (yes)	449 (21.8)	131 (21.1)	136 (19.0)	182 (24.9)	
CHF (yes)	360 (16.7)	102 (17.6)	121 (12.6)	137 (19.6)	
Hyperlipidemia (yes)	1731 (85.4)	549 (81.9)	589 (84.9)	593 (88.7)	
Hypertension (yes)	1639 (76.9)	507 (72.0)	563 (79.2)	569 (78.7)	
DM (yes)	792 (31.6)	241 (30.3)	279 (32.2)	272 (32.2)	
HbA1C, mean ± SD	6.07 (1.40)	5.94 (1.37)	6.02 (1.27)	6.21 (1.52)	
BUN mmol/L, mean ± SD	6.29 (3.08)	5.71 (2.75)	6.24 (3.28)	6.80 (3.06)	
Ca mmol/L, mean ± SD	2.36 (0.11)	2.37 (0.12)	2.35 (0.12)	2.36 (0.11)	
UA *μ*mol/L, mean ± SD	340.40 (100.49)	335.61 (106.48)	325.57 (94.00)	357.64 (98.65)	
Cr *μ*mol/L (mean (SD))	89.87 (60.07)	84.70 (49.40)	88.15 (61.37)	95.62 (66.13)	
Na mmol/L, mean ± SD	139.20 (2.88)	139.36 (2.62)	139.03 (3.28)	139.23 (2.70)	

*Note:* MAR values were multiplied by 100 for presentation purposes. Data are presented as mean ± standard deviation (SD), median (interquartile range, IQR), or number of cases (percentage, %). Comparisons across groups were performed using one‐way ANOVA for normally distributed continuous variables, Kruskal–Wallis H test for non‐normally distributed continuous variables, and the chi‐square (*χ* ^2^) test for categorical variables. *N* reflected the study sample, whereas percentages reflect the proportion of survey data. Pearson′s *χ* ^2^: Rao and Scott adjustment. *n* (weighted %) for categorical variables.

Abbreviations: BMI, body mass index; BUN, blood urea nitrogen; Ca, calcium; CHF, congestive heart failure; Cr, serum creatinine; DM, diabetes mellitus; HbA1c, glycated hemoglobin; IQR, interquartile range; MAR, monocyte‐to‐albumin ratio; Na, sodium; PIR, poverty‐income ratio; SD, standard deviation; UA, uric acid.

^a^Median (IQR) for continuous variables.

^b^Design‐based Kruskal–Wallis test.

To address potential selection bias, missing albumin values in NHANES—primarily resulting from nonattendance at the Mobile Examination Center or refusal of venipuncture [31]—did not support complete random missingness based on baseline comparisons (Table S4) and Little′s MCAR test (*χ* ^2^ = 45,868.8357, *p* < 0.05; Table S5). Given that MAR cannot be verified from the data alone, a sensitivity analysis was conducted under a conservative MNAR assumption [32], wherein missing values were imputed at the clinical lower limit (35 g/L). This approach assumes that missingness reflects poorer nutritional status, thereby evaluating the robustness of the primary findings.

Baseline features were summarized overall and across MAR tertiles. Depending on distribution, normally distributed continuous variables are presented as means ± standard deviation (SD), whereas non‐normally distributed variables are presented as medians with interquartile ranges (IQR). Categorical variables are expressed as frequencies and percentages (%). Comparisons of continuous variables were made using the *t*‐test (for normally distributed data) or the Mann–Whitney *U* test (for non‐normally distributed data), whereas categorical variables were compared using the chi‐square test.

MAR values were relatively small and right‐skewed. For interpretability and to avoid excessive decimal places, MAR was rescaled by multiplying by 100 (MAR × 100). Given the right‐skewed distribution of MAR values, a log2 transformation was applied to these values for scale conversion before undergoing RCS analysis. Effect estimates are reported as the hazard/risk associated with a 1‐unit increase in log₂(MAR × 100), which corresponds to an approximate doubling of the rescaled MAR × 100 value, considering it both as a continuous variable and a categorical variable (< 1.19, 1.19–1.62, and ≥ 1.62), with the lowest tertile (T1) set as the reference group. This categorization (tertiles) was chosen to align with established methodological precedents in MAR‐related epidemiological research, which not only balances statistical power with clinical interpretability but also helps to elucidate potential nonlinear relationships between MAR and mortality [33, 34]. Participants were classified into binary classification according to cutoff values determined by the distribution of MAR within the study cohort. Multiple imputation method (five replicates, using the Markov chain Monte Carlo approach) was employed to handle covariates with missing values (the proportion of missing data for each covariate is summarized in Table S6) [35].

Multivariable‐adjusted Cox regression models were utilized across all analyses to ascertain hazard ratios (HRs) and the corresponding 95% confidence intervals (CIs). Because the proportional hazards assumption for age, smoke status, and drink status was violated (*p* < 0.05 in the Schoenfeld residuals test), a stratified Cox proportional hazards model was employed (Table S7). As shown in Figure S1, the residuals for all covariates were distributed randomly around zero without a discernible trend over time. This visual inspection was further supported by the global statistical test (*p* = 0.158), confirming the validity of the Cox model. Four progressive adjustment models were applied as follows: The association between MAR and all‐cause mortality was assessed using multivariable Cox proportional hazards models. Four models were established: The crude model was unadjusted; Model 1 adjusted for demographic variables (age, gender, race, education level, poverty‐to‐income ratio, and BMI); Model 2 further refined the common clinical risk factors and comorbidities based on Model 1 (including smoke status, drink status, congestive heart failure, hyperlipidemia, hypertension, and diabetes); and Model 3 was fully adjusted for all covariates in Model 2 along with laboratory parameters, including BUN, UA, Cr, and TC. To investigate the dose–response relationship, RCS with four knots positioned at the 5th, 35th, 65th, and 95th percentiles were fitted within the framework of the Cox model. Kaplan–Meier survival curves were generated to visualize the survival disparities among MAR tertiles, and the log‐rank test was employed for comparison.

Predictive performance was evaluated using the area under the receiver operating characteristic curve (AUC). The DeLong test was employed to compare AUCs between the two models. Additionally, the integrated discrimination improvement (IDI) and continuous/net reclassification improvement (NRI) indices were calculated to quantify improvements in risk discrimination and reclassification accuracy. Categorical NRI was calculated using predefined risk strata of < 10%, 10%–20%, and ≥ 20%. The Youden index in ROC curve analysis was used to determine the optimal cutoff value for MAR (Figure S2), to perform sensitivity analysis by removing outliers in RCS analysis. In addition, a simplified model (smoking status, alcohol consumption, diabetes mellitus, and hypertension) and a comprehensive model (including age, gender, cardiovascular disease, and eGFR) were employed to validate the incremental predictive value of MAR for all‐cause mortality in stroke patients. Subgroup analyses and interaction analyses were conducted, stratified by age, sex, race, hypertension, diabetes, and congestive heart failure.

All statistical tests were two‐sided, with the significance threshold set at *p* < 0.05. Analyses were performed using R software (Version 4.2.2, R Foundation; http://www.R-project.org) and the Free Statistics Analysis Platform (Version 2.3.1, Beijing, China, http://www.clinicalscientists.cn/freestatistic).

## 3. Results

### 3.1. Population Characteristics of the Study Cohort

This study encompassed a total of 2022 participants, including 1016 males (50.2%), with a mean age of 66.9 ± 13.3 years (Table 1). When describing the MAR data using categorical variables (tertiles), it was found that high MAR participants who experienced stroke were generally older, were obese, and had a history of smoking. Additionally, individuals with high MAR exhibited a significantly elevated comorbidity burden, especially hypertension (76.9%) and myocardial infarction (21.8%). Significant disparities were also noted in renal function and electrolyte balance parameters, and all comparisons reached statistical significance (*p* < 0.05). As illustrated in Figure S3, patients who experienced all‐cause mortality (Group 1) exhibited significantly higher levels of MAR (Panel A) and monocyte count (Panel C), along with lower albumin levels (Panel B) (all *p* < 0.001).

### 3.2. RCS Regression Analysis

**Figure 2:**
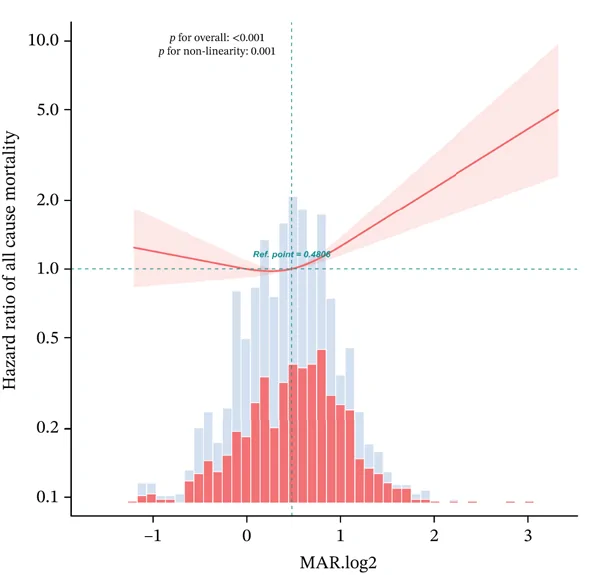
Restricted cubic spline regression of MAR and all‐cause mortality risk in stroke patients. Note: MAR was calculated as (monocyte count/albumin) × 100 and log2‐transformed to address right‐skewness in regression analyses. Solid red line is adjusted hazard ratio (HR); pink shadow is 95% confidence interval (CI). Four knots were placed at the 5th, 35th, 65th, and 95th percentiles. *p* for nonlinearity = 0.001. Adjusted for age, sex, race, poverty‐income ratio, education, smoking, alcohol consumption, BMI, CHF, CHD, hyperlipidemia, hypertension, diabetes, BUN, creatinine, total cholesterol, and uric acid.

RCS regression analysis revealed a nonlinear association between MAR and all‐cause mortality (*p* for nonlinearity = 0.001) (Figure 2). An inflection point was identified at MAR = 1.39 (original value), where the HR equaled 1.0. For MAR values below 1.39, the HR was less than 1.0; however, this apparent inverse association was not statistically significant (HR = 0.802, 95% CI: 0.580–1.109, *p* = 0.182). Conversely, for MAR values ≥ 1.39, the HR was greater than 1.0 and increased monotonically with higher MAR levels, indicating a significant positive association with mortality risk (HR = 1.92, 95% CI: 1.496–2.463, *p* < 0.001) (Table S8).

### 3.3. Independent Association Between MAR and All‐Cause Mortality

We performed four models (crude model was unadjusted). Model 1 adjusted for age, sex, race, PIR, education, smoking status, alcohol consumption status, and BMI. Model 2 further adjusted for comorbidities CHF, hyperlipidemia, hypertension, CHD, and DM. Model 3 further adjusted for BUN, Cr, UA, and TC to investigate the independent association between the MAR and all‐cause mortality in individuals with stroke (Table 2). As a continuous variable, in the fully adjusted Model 3, each 100‐unit increase in MAR (analyzed on a continuous linear scale after multiplying by 100) was independently associated with all‐cause mortality (HR = 1.35; 95% CI: 1.21–1.50; *p* < 0.001). When categorizing into MAR tertile, in the fully adjusted Model 3, there was still a significant association between MAR and all‐cause mortality; the risk of mortality in the T3 group was significantly higher compared with that in the T1 group (T3: HR = 1.32, 95% CI: 1.12~1.55, *p* = 0.001). The values of *p* for trend in all four models were both < 0.001. No statistically significant results were observed in either the etiology‐specific Cox models for CVD or cerebrovascular diseases (Tables S9, S10). Furthermore, to further explore the independent association between MAR and all‐cause mortality, we compared it with other systemic inflammatory markers. The results demonstrated that the assessment efficacy of MAR was not inferior to that of conventional indicators (Figure S4 and Table S11).

**Table 2:** Multivariable Cox regression analysis association between MAR and all‐cause mortality.

MAR (×100)	HR									
	No.	*n* (%)	Crude model	*p* value	Model 1	*p* value	Model 2	*p* value	Model 3	*p* value
Continuous,	2022	985 (48.7)	1.61 (1.48~1.76)	< 0.001	1.40 (1.26~1.55)	< 0.001	1.41 (1.27~1.56)	< 0.001	1.35 (1.21~1.50)	< 0.001
Tertile										
T1 (< 1.19)	649	289 (44.5)	1 (Ref)		1 (Ref)		1 (Ref)		1 (Ref)	
T2 (1.19‐1.62)	696	307 (44.1)	1.16 (0.98~1.36)	0.079	1.12 (0.95~1.32)	0.194	1.11 (0.94~1.31)	0.203	1.05 (0.89~1.24)	0.551
T3 (≥ 1.62)	677	389 (57.5)	1.75 (1.5~2.04)	< 0.001	1.40 (1.19~1.65)	< 0.001	1.39 (1.18~1.63)	< 0.001	1.32 (1.12~1.55)	0.001
*p* for trend				< 0.001		< 0.001		< 0.001		0.001

*Note:* Crude model: No adjustment was made for any covariate. Model 1 was adjusted for age, sex, race, PIR, education, smoking status, alcohol consumption, and BMI; Model 2 was adjusted for Model 1 + CHF, CHD, hyperlipidemia, hypertension, and DM; Model 3 was adjusted for Model 1 + Model 2 + BUN, Cr, TC, and UA. MAR values were multiplied by 100 for presentation purposes. HRs represent the effect per 1‐unit increase in MAR (×100).

Abbreviations: BMI, body mass index; BUN, blood urea nitrogen; CHD, coronary heart disease; CHF, congestive heart failure; Cr, creatinine; DM, diabetes mellitus; HR, hazard ratio; MAR, monocyte‐to‐albumin ratio; PIR, poverty income ratio; TC, total cholesterol; UA, uric acid; 95% CI, 95% confidence interval.

### 3.4. Kaplan–Meier Survival Curve Analysis

**Figure 3:**
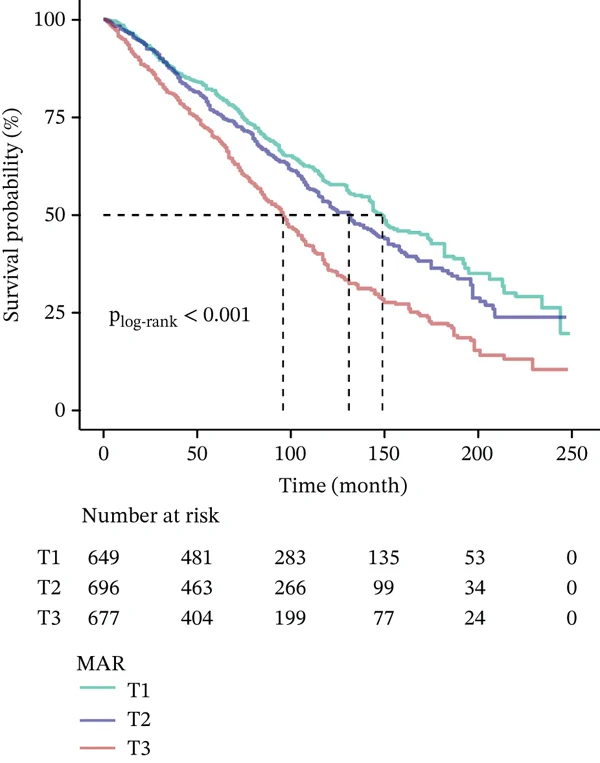
Kaplan–Meier survival curves for all‐cause mortality according to tertiles of MAR. Note: The median survival time was 152 months (95% CI: 140–164) for T1, 138 months (95% CI: 126–150) for T2, and 98 months (95% CI: 88–108) for T3. Group T1 (light green line) represents MAR < 1.19, Group T2 (dark blue line) represents 1.19 ≤ MAR<1.62, and Group T3 (red line) represents MAR ≥ 1.62. Comparison among the three groups was performed using the log‐rank test, revealing a statistically significant difference (*p* < 0.001). The table below the survival curves indicates the number of subjects at risk at each time point. Comparison by log‐rank test, *p* < 0.001.

The Kaplan–Meier curve (Figure 3) illustrates that individuals with elevated MAR(T3) exhibit lower survival probability over a 20‐year follow‐up (median: 7.6 years) period in comparison to those with lower MAR levels(T1) in stroke populations (log‐rank *p* < 0.001).

### 3.5. Incremental Predictive Value of MAR

**Figure 4:**
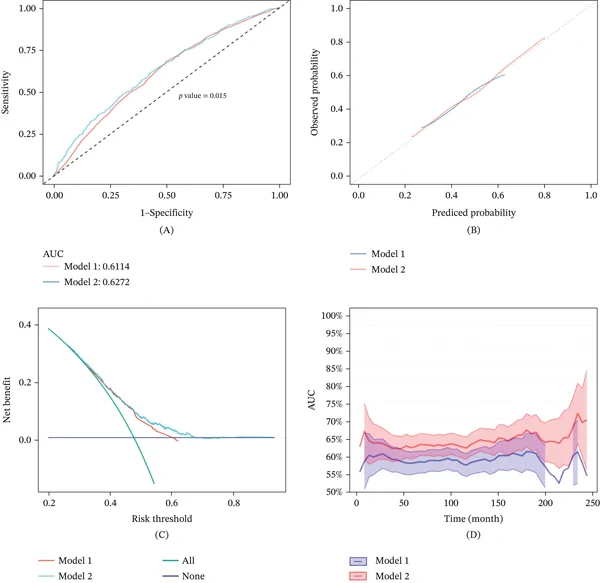
Composite evaluation of predictive performance for Model 1 and Model 2. Note: Model 1 includes smoking, alcohol consumption, hypertension, and diabetes; Model 2 includes Model 1 plus MAR. Panel A is a receiver operating characteristic (ROC) curve that shows that Model 2 has a significantly higher area under the curve (AUC: 0.6272 vs. 0.6114, *p* = 0.015) than Model 1, indicating superior discrimination between events and nonevents. Panel B is a calibration plot that demonstrates better alignment between predicted probabilities and observed outcomes for Model 2 (red line), with its curve closer to the ideal 45° diagonal than that of Model 1 (blue line), reflecting more accurate risk estimation. Panel C is a decision curve analysis (DCA) that reveals that Model 2 yields higher net benefit than Model 1 and the “treat all” strategy across most clinically relevant risk thresholds, confirming enhanced clinical utility. Panel D is a time‐dependent AUC that illustrates that Model 2 (red line with shaded 95% confidence interval) consistently outperforms Model 1 (blue line) over 250 months of follow‐up, with both models maintaining stable predictive performance.

Baseline Model 1 (adjusted for smoking, alcohol use, hypertension, and diabetes) demonstrated moderate discriminability for all‐cause mortality (AUC 0.6114, 95% CI: 0.5871–0.6358). While the addition of MAR in Model 2 resulted in a statistically significant but modest improvement in discrimination (AUC 0.6272, 95% CI: 0.603–0.6513; DeLong test *Δ*AUC = 0.0158, 95% CI: 0.606–0.654, *p* = 0.015) (Table 3 and Figure 4A), the absolute gain was small. Calibration curves (Figure 4B) indicated that Model 2 aligned more closely with the ideal reference line than Model 1 across predicted probability ranges. Decision curve analysis (Figure 4C) demonstrated that Model 2 conferred a higher net benefit than Model 1 and the “treat all” strategy across thresholds of 0.2–0.8, although the absolute increase in net benefit was marginal. Time‐dependent AUC analysis over 250 months (Figure 4D and Table S12) revealed consistently higher values for Model 2 (range: 0.60–0.65) compared to Model 1 (range: 0.58–0.63), with stable performance observed for both models. Reclassification metrics (Table 3) supported the incremental contribution of MAR, yielding an IDI of 0.0129 (95% CI: 0.0080–0.0179, *p* < 0.001), a continuous NRI of 0.208 (95% CI: 0.1218–0.2943, *p* < 0.001), and a categorical NRI of 0.0286 (95% CI: 0.0008–0.0564, *p* = 0.044).

**Table 3:** Changes in predictive performance after adding MAR to the conventional risk model.

Measure	BaseModel^a^	BaseModel + MAR	Improvement	95% CI	*p* value
AUC/C‐statistic	0.6114	0.6272	0.0158	(0.606, 0.654)	0.015
IDI	—	—	0.0129	(0.008, 0.0179)	< 0.001
NRI (continuous)	—	—	0.208	(0.1218, 0.2943)	< 0.001
NRI (category)	—	—	0.0286	(8e‐04, 0.0564)	0.044

*Note:* For the continuous NRI, no predefined risk cutoffs were used. For the categorical NRI, risk strata were defined as < 10%, 10%–20%, and ≥ 20% based on clinical relevance. Statistical significance was determined by the DeLong test for AUC comparison and Z‐test for IDI/NRI. The categorical NRI indicator demonstrated a statistically significant improvement in risk reclassification (*p* = 0.044), with an improvement rate of 2.86% (NRI = 0.0286).

Abbreviations: AUC, area under the receiver operating characteristic curve; IDI, integrated discrimination improvement; NRI, net reclassification improvement.

^a^Base model included traditional risk factors: smoking, alcohol consumption, hypertension, and diabetes mellitus.

### 3.6. Subgroup Analysis and Sensitivity Analysis

**Figure 5:**
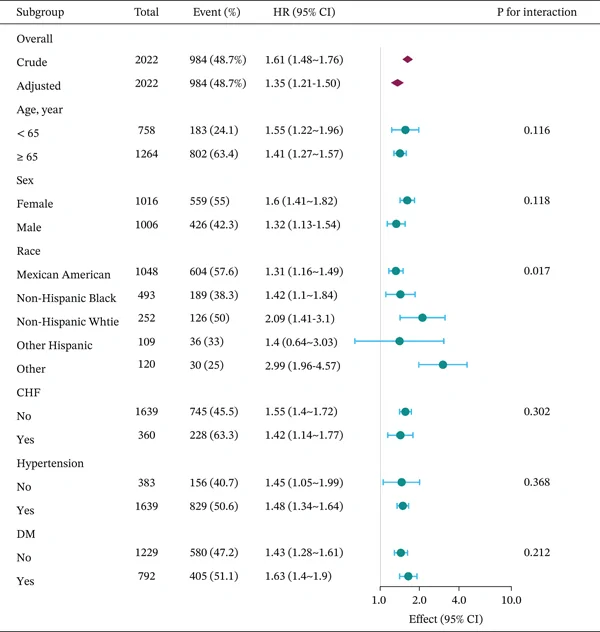
Subgroup analyses of the association between MAR and all‐cause mortality. Note: The forest plot illustrates the subgroup analyses of the association between MAR and all‐cause mortality. HRs (95% CIs) were derived from multivariable Cox regression models adjusted for age, sex, race, and relevant comorbidities. The size of the blue dot represents the point estimate of each subgroup in the overall analysis. *p* for interaction was calculated to test for effect modification across subgroups.

To evaluate the consistency of the association between MAR and all‐cause mortality, we conducted stratified analyses across various clinical subgroups (Figure 5). Overall, after full adjustment, each unit increase in MAR was independently associated with all‐cause mortality (HR = 1.35, 95% CI: 1.21–1.50). Subgroup analyses demonstrated that this association remained robust across different age strata (< 65 and ≥ 65 years), sex, and comorbidity status (CHF, hypertension, and DM) (all *p* for interaction > 0.05 except race subgroup). To assess the robustness of our findings, we also evaluated MAR as a binary using the Youden index‐derived cutoff (1.518). Even under this dichotomized approach, MAR remained significantly associated with increased mortality risk (Table S13). After excluding extreme values (the 0.1st–99th percentiles of MAR distribution), MAR exhibited a significant nonlinear dose–response relationship with all‐cause mortality (Figure S5). Sensitivity analysis under a conservative MNAR scenario for albumin (imputing missing values as 35 g/L) yielded consistent effect estimates for the MAR–mortality association (HR = 1.34, 95% CI: 1.23–1.47; Table S14). Furthermore, incremental prediction analysis adjusted for age, gender, cardiovascular disease, and renal function (eGFR) within a comprehensive model demonstrated an improvement in the risk discrimination capacity of MAR (IDI = 0.0063, *p* < 0.001; continuous NRI = 0.1877, *p* < 0.001; Table S15 and Figures S6).

## 4. Discussion

In this nationally representative cohort of stroke survivors, MAR was independently associated with long‐term all‐cause mortality and displayed a robust nonlinear dose–response relationship, with consistent effects across diverse clinical subgroups. In supplemental analyses, MAR demonstrated statistical utility in improving risk discrimination when added to conventional risk factors; however, these findings should be interpreted as exploratory given the observational design. Rather than a tool for clinical decision‐making, MAR appears to reflect the pathophysiological nexus of inflammation and nutrition in this cohort.

Our findings contribute to the growing body of evidence regarding MAR in cardiovascular health. While prior studies have established MAR as a correlate of short‐term prognosis in acute ischemic stroke [36] and heart failure [37], these investigations were often constrained by brief follow‐up periods. By utilizing the NHANES‐linked mortality data with extended follow‐up, our study provides additional insights into the persistence of this association beyond the acute phase. Unlike short‐term outcomes, which are heavily influenced by initial clinical events, the long‐term mortality observed here likely reflects the cumulative burden of systemic inflammation and chronic malnutrition. The consistency of our results with studies in other cardiovascular contexts lends epidemiological support to MAR′s relevance in long‐term survival [37, 38].

The biological plausibility of our findings is rooted in the synergistic “inflammation‐malnutrition cycle.” Elevated monocyte counts promote foam cell formation and release pro‐inflammatory cytokines (e.g., TNF‐*α* and IL‐1*β*), which exacerbate endothelial dysfunction and suppress eNOS activity, thereby compromising cerebral perfusion [39, 40]. Concurrently, these cytokines inhibit hepatic albumin synthesis. Hypoalbuminemia, in turn, impairs antioxidant defenses—particularly the free radical scavenging capacity of the Cys‐34 sulfhydryl group—leading to lipid peroxidation and blood–brain barrier disruption [41, 42]. This self‐perpetuating cycle creates a catabolic state that heightens vulnerability to recurrent vascular events and accelerates mortality [43]. Unlike single‐dimension markers (e.g., NLR focusing solely on inflammation or albumin alone) [44], MAR′s composite nature captures this dynamic interplay, explaining its superior performance in our incremental value analysis.

In fully adjusted models, MAR exhibited a robust association with all‐cause mortality (HR = 1.35). This effect size was broadly comparable to that of other inflammatory indices, such as NLR (HR = 1.12) and PLR (HR = 1.1), which showed attenuated magnitudes upon multivariate adjustment. ROC analyses further characterized these relationships, revealing similar discriminatory capacities among MAR (AUC = 0.571), NLR (AUC = 0.592), and SII (AUC = 0.563), although MAR modestly outperformed serum albumin alone (AUC = 0.493). These data suggest that MAR captures a synergistic inflammatory–nutritional axis [43]; however, its incremental discriminatory value over established composite indices appears marginal within this cohort. While MAR was derived from routine laboratory parameters, facilitating its use in epidemiological datasets, these findings do not yet support its role as a standalone clinical tool. Regarding cause‐specific outcomes, MAR demonstrated a consistent but nonsignificant positive trend for both CVD and cerebrovascular mortality. The lack of statistical significance likely reflects limited power due to the relatively few specific‐cause events [45], rather than a true absence of association, reinforcing MAR′s relevance to overall mortality risk in stroke survivors. Finally, in the incremental prediction analysis, the consistency of our findings across both parsimonious and fully adjusted models supports the statistical robustness of the MAR′s incremental contribution, even in settings with limited clinical detail [46].

Several features of this study enhance the interpretation of our findings. Methodologically, the use of a large, nationally representative cohort of stroke survivors (*n* = 2022) provided adequate statistical power. Crucially, the primary association between MAR and mortality was established using a fully adjusted model incorporating comprehensive demographic, lifestyle, and clinical covariates, ensuring rigorous control for potential confounders. Practically, MAR is derived from routine complete blood counts and albumin assays, facilitating its incorporation into large‐scale epidemiological datasets without the need for specialized assays (e.g., genomic sequencing or circulating microRNAs). Furthermore, our findings demonstrated robustness across rigorous sensitivity analyses, including the application of the Youden index‐derived cutoff and sequential exclusion of extreme values. Notably, while our primary analysis utilized a fully adjusted model, supplementary analyses exploring incremental value employed a parsimonious baseline model (incorporating hypertension, diabetes, smoking, and alcohol consumption) to simulate resource‐limited settings.

However, these strengths are tempered by certain limitations inherent to the NHANES design. First, our primary analyses did not incorporate survey weights. In weighted analyses (Table S3), the association was attenuated and no longer reached statistical significance, likely reflecting reduced precision due to weighted variance inflation in a sample of 2022 stroke survivors. However, the direction and magnitude of the point estimate remained consistent, and the lack of significance in the weighted analysis likely reflects reduced statistical power of the primary finding. This indicates that this trade‐off did not significantly affect our estimates of internal effects but may have limited the generalizability of the findings to the entire US population. Second, while the addition of MAR resulted in a statistically significant improvement in model discrimination (*p* = 0.015), the absolute increment in AUC was modest (from 0.6114 to 0.6272). This underscores that long‐term mortality in stroke survivors is driven by multifactorial processes that cannot be fully captured by a single biomarker. We acknowledge that the parsimonious baseline model—intentionally designed to simulate pragmatic, resource‐limited screening scenarios—may provide an upper bound estimate of MAR′s incremental value. Nevertheless, sensitivity analyses incorporating established predictors such as age, sex, renal function, and comorbidities demonstrated that MAR remains valuable for assessing all‐cause mortality among stroke survivors (Table S15 and Figure S6), but its clinical implementation requires caution given the modest magnitude of improvement, and it should not replace established clinical risk assessment tools. Third, residual confounding from unmeasured clinical variables persists. A major limitation of the NHANES database is the lack of stroke‐specific granularity. Notably, stroke diagnoses were based on self‐report without imaging confirmation, precluding validation of etiological subtypes or assessment of acute severity (e.g., NIHSS scores and infarct volumes). Furthermore, critical determinants of long‐term prognosis—including reperfusion therapy, recurrent stroke events, postdischarge rehabilitation intensity, and specific secondary prevention medication use—were not available. The single baseline assessment of MAR also failed to capture temporal metabolic fluctuations during follow‐up. Given the observational design and these substantial unmeasured confounders, the observed association should be interpreted as exploratory and should not be construed as evidence of a causal relationship between MAR and mortality. While sensitivity analyses (e.g., E‐value, point estimate = 2.04; Figure S7) suggested that our findings were robust to moderate confounding, these unmeasured factors limit the ability to infer causality. Additionally, baseline data were not missing completely at random (Little′s test, *p* < 0.05), which may introduce selection bias beyond measured covariates. Future prospective, multicenter validation studies—ideally incorporating serial biomarker measurements and adjudicated clinical endpoints—are warranted to corroborate these findings in phenotypically diverse cohorts. Such studies should also investigate whether targeted interventions addressing the underlying inflammation–nutrition axis can mitigate the excess mortality risk associated with elevated MAR, thereby translating this epidemiological association into tangible clinical benefits.

## 5. Conclusion

In conclusion, this nationally representative cohort study demonstrates that elevated MAR is independently associated with long‐term all‐cause mortality among stroke survivors. While incremental improvements in model performance were observed, the modest absolute gains underscore that MAR currently serves best as a research metric for assessing combined burden of systemic inflammation and nutritional decline in epidemiological contexts, rather than a tool for individualized clinical risk stratification. Future prospective studies are warranted to validate these findings and elucidate MAR′s potential role in guiding targeted interventions.

## Author Contributions

The authors′ responsibilities were as follows: Jia Wang designed the research and wrote the first draft of the paper; Yue Wu conducted analyses and revised the manuscript; and Jing Xia conducted analyses and revised the manuscript.

## Funding

No funding was received for this manuscript.

## Disclosure

All authors read and approved the final manuscript and approved the final submitted version.

## Ethics Statement

All participants provided written informed consent, and study procedures were approved by the National Center for Health Statistics Research Ethics Review Board (Protocol Number: Protocol #98‐12, Protocol #2005‐06, and Protocol #2011‐17).

## Consent

The authors have nothing to report.

## Conflicts of Interest

The authors declare no conflicts of interest.

## Supporting information


**Supporting Information** Additional supporting information can be found online in the Supporting Information section. Table S1: Comparative overview of the present study and previously published NHANES‐based investigations on inflammation–nutrition biomarkers in stroke populations. Supporting Information 2. Table S2: Variance inflation factors (VIFs) for all covariates assessed in the multivariable Cox regression models. Supporting Information 3. Text S1: Detailed description of laboratory assay methodologies and quality assurance procedures for NHANES biomarkers (1999–2018). Supporting Information 4. Table S3: Weighted and unweighted Cox regression analyses examining the association between MAR and all‐cause mortality across crude and sequentially adjusted models. Supporting Information 5. Table S4: Baseline demographic and clinical comparisons between participants included in the final analysis and those excluded due to missing albumin values. Supporting Information 6. Table S5: Little′s Missing Completely at Random (MCAR) test results for variables included in the imputation and survival models. Supporting Information 7. Table S6: Frequency and percentage distribution of missing values for all study variables. Supporting Information 8. Table S7: Schoenfeld residual tests for the proportional hazards assumption in the Cox regression models, stratified by follow‐up time. Supporting Information 9. Figure S1: Time‐dependent Schoenfeld residual plot for MAR tertiles. Supporting Information 10. Figure S2: Receiver operating characteristic (ROC) curve for MAR in predicting all‐cause mortality, identifying the optimal cutoff value via the Youden index. Supporting Information 11. Figure S3: Bar graphs comparing mean values of MAR, serum albumin, and monocyte count between survivors and nonsurvivors. Supporting Information 12. Table S8: Threshold effect analysis of MAR on all‐cause mortality using piecewise Cox regression. Supporting Information 13. Table S9: Multivariable Cox regression analyses evaluating the association between MAR and cardiovascular disease (CVD) mortality. Supporting Information 14. Table S10: Multivariable Cox regression analyses evaluating the association between MAR and cerebrovascular disease‐specific mortality. Supporting Information 15. Table S11: Head‐to‐head comparative analysis of MAR versus conventional inflammatory and nutritional biomarkers (NLR, PLR, SII, PNI, albumin, and monocyte count) in predicting all‐cause mortality. Supporting Information 16. Figure S4: ROC curves comparing the discriminative performance of seven biomarkers for long‐term mortality prediction. Supporting Information 17. Table S12: Incremental predictive value of MAR when added to conventional risk prediction models for all‐cause mortality at 3‐, 5‐, 8‐, 10‐, 15‐, and 20‐year follow‐up intervals. Supporting Information 18. Table S13: Stratified multivariable Cox regression analyses for MAR utilizing both tertile‐based and Youden‐index‐derived cutoff values. Supporting Information 19. Figure S5: Restricted cubic spline analysis demonstrating the nonlinear dose–response relationship between MAR and all‐cause mortality risk after outlier exclusion. Supporting Information 20. Table S14: Sensitivity analysis addressing potential missing not at random (MNAR) bias in albumin measurements through best case scenario imputation. Supporting Information 21. Table S15: Sensitivity analysis of MAR′s incremental predictive utility within a fully adjusted baseline model incorporating cardiometabolic and renal function covariates. Supporting Information 22. Figure S6: Comprehensive model performance evaluation comparing baseline and MAR‐augmented models. Supporting Information 23. Figure S7: Bias contour plot illustrating the potential impact of residual confounding on the observed exposure–outcome association.

## Data Availability

NHANES data described in this manuscript are available at https://wwwn.cdc.gov/nchs/nhanes/.
