# Supplementary material for: Monocyte‐to‐Albumin Ratio (MAR) Is Associated With All‐Cause Mortality in Adult Stroke Patients: Evidence From NHANES (1999–2018)
Source: Stroke Res Treat. 2026 Aug 25;2026:5461096. doi: 10.1155/srat/5461096 (PMC13507710; doi:10.1155/srat/5461096)
Supplement: Supplementary file 1 [file SRAT-2026-5461096-s001.docx]

**Table S1** Comparison of the current study with previously published NHANES-based studies on inflammation-nutrition biomarkers in stroke populations.

| **Study (Pmid, year)** | **Biomarker** | **Population** | **Stroke n** | **Median Follow-up** | **Endpoints** | **Key Finding in Stroke Subset** | **Distinct Contribution** |
| --- | --- | --- | --- | --- | --- | --- | --- |
| [41048951]  (2025) | NLR | Stroke survivors | 1,229 | 6.41 y | All-cause, CVD mortality | Q4 vs Q1: all-cause HR=1.58 | First study to reveal J-shaped NLR-mortality association in stroke survivors |
| [40520535 ]  (2025) | NPAR | Older stroke survivors | 1,026 | 6.65 y | All-cause, CVD mortality | Q4 vs Q1: all-cause:  HR=1.69 | First study linking NPAR to mortality in community-dwelling older stroke survivors |
| [40155756]  (2025) | NPS | Stroke patients | 1,372 | 5.94 y | All-cause mortality | Group2 vs 0: HR=2.21 | It was demonstrated that NPS exhibits the strongest predictive capacity for all-cause mortality in stroke patients. |
| [41059443]  (2025) | MAR | Diabetes patients | 23,740 | 8.3 y | CVD prevalence +All-cause & CVD mortality | T3 vs T1: all-cause HR=1.25  CVD mortality HR =1.46 | First large-scale study linking MAR to CVD and mortality in general population. |
| [40855352 ]  (2024) | MAR | General population | 15,679 | Cross-sectional | Stroke prevalence | Q4 vs Q1: OR≈2.5× stroke prevalence | MAR outperforms TyG index in predicting stroke prevalence, especially in diabetics |
| **Our Study** | **MAR** | **Stroke survivors** | **2,022** | **7.6 y** | **All-cause mortality** | **HR = 1.35** | **Only study to examine MAR specifically in stroke survivors with up to 20 years of follow‑up (vs. previous MAR studies in diabetes or general population)** |

**Notes:** Biomarker: NLR, NPAR, NPS, MAR. Population: Refers to the specific characteristics of the study participants as reported in the original literature. Stroke n: Sample size of participants with stroke or stroke survivors in each study. Endpoints: All-cause mortality indicates death from any cause; CVD mortality indicates death specifically due to cardiovascular disease. Key Finding: HR (Hazard Ratio) values represent the risk of the respective mortality endpoint for the highest quartile (Q4) or specific high-risk group compared to the lowest quartile (Q1) or reference group (Group 0), unless otherwise specified. CI denotes Confidence Interval. Distinct Contribution of Our Study: This column highlights the unique aspects of our study compared to existing literature, such as targeting a specific patient population (stroke survivors) or focusing on a novel biomarker (MAR).

**Abbreviations:** NHANES, National Health and Nutrition Examination Survey; MAR, Monocyte-to-Albumin Ratio; NLR, Neutrophil-to-Lymphocyte Ratio; NPAR, Neutrophil-to-Albumin Ratio; NPS, Naples Prognostic Score; HR, Hazard Ratio; CI, Confidence Interval; CVD, Cardiovascular Disease.

**Table S2** Variance inflation factors(VIFs) for covariates included.

| **variable** | **coeff** | **Change.percentage** | **GVIF** | **DF** | **GVIF^(1/(2*Df))** | **collinearity** | **select** | **select.VIF** |
| --- | --- | --- | --- | --- | --- | --- | --- | --- |
| Crude | 0.43 | Ref. | 1.186 | 1 | 1.089 | 0 | Ref. | Ref. |
| Age | 0.29 | -32.5 | 1.669 | 1 | 1.292 | 0 | Yes | Yes |
| Sex | 0.39 | -7.6 | 1.559 | 1 | 1.248 | 0 | No | No |
| Race | 0.39 | -9.4 | 2.476 | 4 | 1.12 | 0 | Yes | Yes |
| Marital status | 0.44 | 3 | 1.308 | 1 | 1.144 | 0 | No | No |
| PIR | 0.42 | -0.4 | 1.428 | 1 | 1.195 | 0 | Yes | Yes |
| Education | 0.43 | 1.6 | 1.725 | 4 | 1.071 | 0 | Yes | Yes |
| Alcohol consumption | 0.51 | 20.5 | 1.438 | 2 | 1.095 | 0 | Yes | Yes |
| Smoke status | 0.43 | 1.1 | 1.956 | 2 | 1.183 | 0 | Yes | Yes |
| BMI, kg/m^2^ | 0.48 | 13.1 | 1.365 | 1 | 1.169 | 0 | Yes | Yes |
| BUN, mmol/l | 0.32 | -24.5 | 2.163 | 1 | 1.471 | 0 | Yes | Yes |
| TC, mmol/L | 0.75 | 75.5 | 1.185 | 1 | 1.089 | 0 | Yes | Yes |
| Cr, umol/l | 0.36 | -14.8 | 1.989 | 1 | 1.41 | 0 | Yes | Yes |
| UA, mmol/l | 0.38 | -10.3 | 1.445 | 1 | 1.202 | 0 | Yes | Yes |
| **HbA1C** | 0.43 | 0.5 | 3.032 | 1.0 | 1.741 | 0.0 | No | **No** |
| **Cl, mmol.l** | 0.42 | -1.7 | 1.268 | 1.0 | 1.126 | 0.0 | No | **No** |
| **Globulin, g/L** | 0.38 | -0.3 | 1.445 | 1.0 | 1.202 | 0.0 | No | **No** |
| **Calcium, mmol/L** | 0.42 | -0.3 | 1.25 | 1.0 | 1.118 | 0.0 | No | **No** |
| **Na_mmol.l** | 0.42 | -0.8 | 1.788 | 1.0 | 1.337 | 0.0 | No | **No** |
| CHF | 0.41 | -2.6 | 1.299 | 1 | 1.14 | 0 | No | No |
| Hyperlipidemia | 0.43 | 0.7 | 1.128 | 1 | 1.062 | 0 | No | No |
| HTN | 0.41 | -3.5 | 1.179 | 1 | 1.086 | 0 | No | No |
| DM | 0.43 | 0.7 | 1.781 | 1 | 1.335 | 0 | No | No |
| Note: All VIF values were less than 5, indicating the absence of problematic multicollinearity.  Note: Abbreviations: BMI, body mass index; BUN, blood urea nitrogen; CHF, congestive heart failure; Cr, creatinine; DM, diabetes mellitus; HDL-C, high-density lipoprotein cholesterol; HTN, hypertension; LDH, lactate dehydrogenase; PIR, poverty-to-income ratio. | | | | | | | | |

**Supplemental Text S1.** Detailed Laboratory Assay Methods for NHANES Biomarker Measurements (1999–2018)

# All laboratory biomarkers included in this study—hemoglobin A1c (HbA1c, %), blood urea nitrogen $\text{(}\text{BUN}\text{,}\text{mg}\text{/}\text{dL}\text{)}$, serum calcium $\text{Ca}\text{,}\text{mg}\text{/}\text{dL}$, serum creatinine ($\text{Cr}\text{,}\text{mg}\text{/}\text{dL}\text{)}$, serum sodium $\text{Na}\text{,}\text{mmol}\text{/}\text{L}$, serum chloride ($\text{Cl}\text{,}\text{mmol}\text{/}\text{L}\text{)}$, and serum globulin (g/L)—were obtained from the National Health and Nutrition Examination Survey (NHANES, 1999–2018 cycles). Blood specimens were collected at the NHANES Mobile Examination Center (MEC) by trained phlebotomists and processed according to standardized protocols established by the Centers for Disease Control and Prevention (CDC), National Center for Health Statistics (NCHS), and the Division of Laboratory Sciences (DLS). Detailed assay procedures are described below.

## Hemoglobin A1c (HbA1c, %), HbA1c was measured in whole blood using ion-exchange high-performance liquid chromatography (HPLC). 1999–2004: Primus CLC330 or CLC385 boronate-affinity HPLC analyzer; 2005–2006: Tosoh A1c 2.2 Plus Glycohemoglobin Analyzer; 2007–2018: Tosoh G7 HPLC Analyzer. All methods were certified by the National Glycohemoglobin Standardization Program (NGSP) and traceable to the Diabetes Control and Complications Trial (DCCT) reference.

## Blood Urea Nitrogen (BUN,mg/dl), BUN was measured in serum using an enzymatic urease-conductometric method on Beckman Coulter Synchron LX20 or UniCel DxC800 chemistry analyzers. The method quantifies urea following urease hydrolysis via conductivity changes.

## Serum Calcium ($\text{Ca}\text{,}\text{mg}\text{/}\text{dL}$),Total serum calcium was measured using an arsenazo III colorimetric assay on Beckman Coulter chemistry analyzers. Values were standardized to NIST SRM 909b reference material.

## Serum Creatinine $\text{(}\text{Cr}\text{,}\text{mg}\text{/}\text{dL}\text{)}$, Serum creatinine was measured using the kinetic Jaffe (alkaline picrate) method on Beckman Coulter or Hitachi chemistry analyzers. Beginning with NHANES 2005–2006, values were calibrated to isotope dilution mass spectrometry (IDMS)–traceable reference standards; earlier-cycle values (1999–2004) were adjusted using NHANES-recommended correction equations to allow pooling across cycles.

## Serum Sodium (Na, mmmo/L) and Chloride (Cl, mmol/L), Sodium and chloride were measured simultaneously using indirect ion-selective electrodes (ISE) integrated into Beckman Coulter or Hitachi chemistry analyzers as part of the standard electrolyte panel.

## Serum Globulin (g/L), Serum globulin was not measured directly, but calculated as:

Globulin = Total Protein − Albumin. Total protein was measured using a biuret colorimetric assay, and albumin was measured using a bromocresol purple (BCP) dye-binding assay, both on Beckman Coulter or Hitachi chemistry analyzers.

Quality Assurance and Quality Control: All NHANES laboratory components followed a rigorous CLIA-compliant QA/QC program, including calibration verification, blinded duplicate specimens, Westgard rule application, and external proficiency testing. Intra-assay and inter-assay coefficients of variation (CVs) were typically <5% for all analytes. Detailed limits of detection (LODs), CVs, and specimen-handling procedures are provided in the NHANES Laboratory/Medical Technologists Procedures Manual, available at: https://wwwn.cdc.gov/nchs/nhanes/default.aspx

All laboratory variables were analyzed as continuous measures in regression models.

**Table S3** Association between MAR and all-cause mortality using weighted and unweighted Cox regression models.

| **Variable** | **Unweighted Model (HR, 95% CI)** | **Weighted Model (HR, 95% CI)** |
| --- | --- | --- |
| MAR (per log2) | 1.31 (1.12~1.52) | 1.47 (0.96, 2.27) |
| Tertile |  |  |
| T1 ( < 1.19) | 1(Ref) | 1(Ref) |
| T2 (1.19-1.62) | 1.12 (0.92~1.36) | 0.97(0.56, 1.68) |
| T3 ( ≥ 1.62) | 1.25 (1.03~1.52) | 1.43(0.81, 2.54) |
| P for Trend | 0.022 | 0.216 |

**Crude model**: no adjustment was made for any covariate.

**Model 1** was adjusted for age, sex, race, PIR, education, smoking status, alcohol consumption, and BMI;

**Model 2** was adjusted for Model1 + CHF, CHD, Hyperlipidemia, Hypertension, and DM;

**Model 3** was adjusted for Model1 + Model2 + BUN, Cr, TC, UA.

**Note:** MAR was calculated as monocyte count (×10⁹/L) divided by serum albumin (g/L) and subsequently multiplied by 100 (MAR×100). The Cox proportional hazards models were presented for both unweighted analyses (primary results) and weighted analyses using NHANES sampling weights (WTMEC2YR for 2003–2018 and 2/10 × WTMEC4YR for 1999–2002) as a sensitivity analysis. HRs for the continuous variable represent the risk associated with a doubling of MAR×100. Tertile cut-off points were derived from the weighted distribution of the study population. P for Trend was calculated using the median value of each tertile as a continuous variable.

**Table S4** Comparison of baseline characteristics between participants included and excluded due to missing albumin.

| **Variables** | **Total (n = 126348)** | **Included (n = 70204)** | **Excluded (n = 56144)** | ***p*** |
| --- | --- | --- | --- | --- |
| Age, Mean ± SD | 49.9 ± 18.5 | 49.7 ± 18.2 | 50.8 ± 19.5 | < 0.001 |
| Sex, n (%) |  |  |  | < 0.001 |
| Male | 32695 (47.8) | 25581 (48.1) | 7114 (46.6) |  |
| Female | 35718 (52.2) | 27558 (51.9) | 8160 (53.4) |  |
| Race, n (%) |  |  |  | < 0.001 |
| Mexican-American | 30214 (44.2) | 23723 (44.6) | 6491 (42.5) |  |
| Spanish-American | 14060 (20.6) | 10644 (20) | 3416 (22.4) |  |
| Non-Spanish white people | 13190 (19.3) | 9837 (18.5) | 3353 (22) |  |
| Non-Spanish black people | 5371 ( 7.9) | 4316 (8.1) | 1055 (6.9) |  |
| Other non-Spanish ethnic groups | 5578 ( 8.2) | 4619 (8.7) | 959 (6.3) |  |
| Marry, n (%) |  |  |  | < 0.001 |
| Married | 39770 (59.9) | 31690 (60.7) | 8080 (57) |  |
| Other | 26619 (40.1) | 20526 (39.3) | 6093 (43) |  |
| PIR, Mean ± SD | 2.5 ± 1.6 | 2.5 ± 1.6 | 2.5 ± 1.6 | < 0.001 |
| Education, n (%) |  |  |  | < 0.001 |
| Less than 9th grade | 9349 (13.7) | 6710 (12.6) | 2639 (17.4) |  |
| 9th–11th grade (no diploma) | 10948 (16.0) | 8100 (15.3) | 2848 (18.7) |  |
| High school graduate / GED | 15754 (23.1) | 12223 (23) | 3531 (23.2) |  |
| Some college or AA degree | 18333 (26.9) | 14811 (27.9) | 3522 (23.2) |  |
| College graduate or higher (≥ Bachelor's) | 13864 (20.3) | 11213 (21.1) | 2651 (17.5) |  |
| Smoke status, n (%) |  |  |  | 0.759 |
| Current smoker | 37023 (54.2) | 28768 (54.2) | 8255 (54.2) |  |
| Former smoker | 17136 (25.1) | 13288 (25) | 3848 (25.3) |  |
| Never smoker | 14160 (20.7) | 11030 (20.8) | 3130 (20.5) |  |
| Drinking status, n (%) |  |  |  | < 0.001 |
| Current drinker | 8835 (15.0) | 7043 (14.7) | 1792 (16.3) |  |
| Former drinker | 10912 (18.5) | 8641 (18) | 2271 (20.7) |  |
| Never drinker | 39262 (66.5) | 32344 (67.3) | 6918 (63) |  |
| BMI kg/m2, Mean ± SD | 28.8 ± 6.7 | 28.9 ± 6.7 | 28.4 ± 6.5 | < 0.001 |
| HbA1c, Mean ± SD | 5.6 ± 1.0 | 5.6 ± 1.0 | 5.8 ± 1.3 | < 0.001 |
| Angina, n (%) | 2101 ( 3.1) | 1569 (3) | 532 (3.5) | < 0.001 |
| Heart.attack, n (%) | 3081 ( 4.5) | 2371 (4.5) | 710 (4.7) | 0.3 |
| Congestive.heart.failure, n (%) | 2305 ( 3.4) | 1715 (3.2) | 590 (3.9) | < 0.001 |
| Hyperlipidemia, n (%) | 45687 (69.5) | 38674 (72.8) | 7013 (55.8) | < 0.001 |
| Hypertension, n (%) | 28388 (41.5) | 22161 (41.7) | 6227 (40.9) | 0.066 |
| DM, n (%) | 11111 (16.8) | 9021 (17.5) | 2090 (14.4) | < 0.001 |
| UA, umol/L, Mean ± SD | 316.0 ± 85.4 | 316.0 ± 85.4 | 333.1 ± NA | 0.842 |
| Na, mmol/L, Mean ± SD | 139.2 ± 2.4 | 139.2 ± 2.4 | 136.0 ± NA | 0.176 |
| Ca, mmoll/L, Mean ± SD | 2.4 ± 0.1 | 2.4 ± 0.1 | 2.3 ± NA | 0.351 |

**Abbreviations:** NHANES, National Health and Nutrition Examination Survey; PIR, Poverty Income Ratio; HbA1C, Hemoglobin A1c; UA, Uric Acid; DM, Diabetes Mellitus; SD, Standard Deviation.

**Table S5** The result of little's Missing Completely at Random Test.

| **Chi-square value** | **degree.free** | **p.value** | **missing.patterns** |
| --- | --- | --- | --- |
| 45868.8357 | 5014.0 | < 0.001 | 302 |

Note: Data were sourced from the NHANES database, with a total sample size of 73,983 cases. The examined variables included serum uric acid, serum creatinine, monocyte count, and all-cause mortality.

****Table S6** Frequency and Percentage of Missing Values for Study Variables.**

| **Variables** | **Miss frequency** | **Miss percentage, %** |
| --- | --- | --- |
| Age, year | 0 | 0 |
| Albumin, g/L | 0 | 0 |
| All cause mortality | 0 | 0 |
| Angina | 27 | 1.3353 |
| BMI, kg/m^2^ | 128 | 6.3304 |
| BUN, mmol/L | 0 | 0 |
| Cholesterol, mmol/L | 0 | 0 |
| Congestive heart failure | 23 | 1.1375 |
| Coronary heart disease | 42 | 2.0772 |
| Cr, umol/L | 0 | 0 |
| CVD | 0 | 0 |
| DM | 1 | 0.0495 |
| Drink status | 232 | 11.4738 |
| Education level | 3 | 0.1484 |
| HbA1C | 3 | 0.1484 |
| Heart attack | 11 | 0.544 |
| Hyperlipidemia | 0 | 0 |
| Hypertension | 0 | 0 |
| MAR | 0 | 0 |
| Marrital status | 38 | 1.8793 |
| PIR | 180 | 8.9021 |
| Race | 0 | 0 |
| Sex | 0 | 0 |
| smoke | 2 | 0.0989 |
| Uric acid umol/L | 0 | 0 |

**Note: This table presents the frequency and percentage of missing values for each variable included in the final analysis. A small proportion of missing data was observed for variables including Drink status (11.47%), PIR (8.90%), and BMI (6.33%). All other variables had complete data with no missing values.**

**Table S7** Results of testing the proportional hazards assumption for covariates in the Cox model (using time stratification).

| **Variable** | **chisq** | | | **df** | ***p*** |
| --- | --- | --- | --- | --- | --- |
| Age | | 2.573 | 1.0 | | 0.109 |
| Sex | | 1.202 | 1.0 | | 0.273 |
| Race | | 5.764 | 4.0 | | 0.217 |
| PIR | | 2.69 | 1.0 | | 0.101 |
| BMI, kg.m2 | | 1.74 | 1.0 | | 0.187 |
| Hyperlipidemia | | 3.544 | 1.0 | | 0.06 |
| Hypertension | | 0.891 | 1.0 | | 0.345 |
| DM | | 0.032 | 1.0 | | 0.857 |
| BUN, mmol/L | | 0.045 | 1.0 | | 0.831 |
| Cr, umol/L | | 0.419 | 1.0 | | 0.517 |
| UA, umol/L | | 0.247 | 1.0 | | 0.619 |
| TC, mmol/L | | 0.066 | 1.0 | | 0.797 |
| CHF | | 0.203 | 1.0 | | 0.652 |
| MAR (tertile) | | 0.795 | 1.0 | | 0.373 |
| CHD | | 0.084 | 1.0 | | 0.772 |
| MAR(100 conversion) | | 0.704 | 1.0 | | **0.401** |
| MAR.100.Q3:strata(tgroup) | | 2.066 | 3.0 | | **0.559** |
| GLOBAL | | 28.548 | 22.0 | | **0.158** |

Note: This table presents the results of testing the proportional hazards (PH) assumption for all covariates included in the Cox model using time stratification. Bold text indicates the test result for the primary exposure variable (MAR top tertile) after time stratification. The global test (GLOBAL) yielded a P-value of 0.158 (> 0.05), suggesting that the PH assumption holds for all variables in the model after accounting for time-varying effects.

Abbreviations: BMI, body mass index; BUN, blood urea nitrogen; CHF, congestive heart failure; CHD, coronary heart disease; Cr, creatinine; DM, diabetes mellitus; HDL-C, high-density lipoprotein cholesterol; HTN, hypertension; LDH, lactate dehydrogenase; MAR, monocyte-to-albumin ratio; PIR, poverty-to-income ratio; TC, total cholesterol; UA, uric acid.

**Figure S1** Time - dependent test of Proportional Hazards (PH) Assumption for MAR (tertile) after Stratification (Based on Schoenfeld Residuals).

**Note:** The plot shows the time - dependent test of the proportional hazards (PH) assumption for the MAR group (tertile) using Schoenfeld residuals. X - axis (Time): Follow - up time (unit: month); Y - axis: Time - dependent regression coefficient (β(t)) of the MAR(tertile) groupp; Red solid line: Smoothed fitting curve of the regression coefficient, intuitively showing the time - trend of the effect; Red dashed lines: 95% confidence interval of the fitting curve; Gray dashed line (Reference line for null effect): Null - effect reference line (corresponding to HR = 1, i.e., no additional mortality risk). Results indicate that the regression coefficient (β(t)) of the MAR (tertile) group does not show a significant deviation from the null - effect line over time (the fitting curve is generally close to the gray dashed line), suggesting that after stratification and time - dependent adjustment), the effect of MAR(tertile) on mortality risk does not severely violate the PH assumption (Global test: chisq = 28.548, P = 0.158, supporting the validity of the PH assumption).

**Figure S2. Receiver Operating Characteristic (ROC) curve analysis for the prediction of all-cause mortality using the MAR index (multiplied by 100).**

Note: The area under the curve (AUC) is 0.571 (95% confidence interval [CI]: 0.546–0.596). The optimal cutoff value of **1.518** was determined based on the Youden index, corresponding to a sensitivity of 65.2% and a specificity of 48.0%.

****Figure S3.** Differences in the distribution of MAR (A), albumin (B), and monocyte count (C) among populations.**

Note: This figure illustrates the mean values and standard errors (SE) of three key biomarkers between participants who experienced all-cause mortality (Group 1, blue) and those who did not (Group 0, red). Panel A shows that the MAR index was significantly higher in the mortality group compared to the non-mortality group (P < 0.001). Panel B demonstrates that serum albumin levels were significantly lower in the mortality group (P < 0.001). Panel C indicates that monocyte counts (×10⁹/L) were significantly elevated in the mortality group compared to the non-mortality group (P < 0.001). Error bars represent standard error (SE). Different lowercase letters (a, b) above the bars indicate statistically significant differences between groups (P < 0.05, post-hoc test).

**Table S8** Threshold Effect Analysis of MAR on the Risk of All-Cause Mortality.

| ****Statistical Analysis** N** | ****Value**** | ****P-value**** |
| --- | --- | --- |
| Inflection Point | 1.39 | NA |
| < 1.39 886 | 0.802 (0.58–1.109) | 0.182 |
| ≥ 1.39 1136 | **1.92 (1.496–2.463)** | **<0.001** |
| Likelihood Ratio Test (LRT) |  | **0.001** |
| Non-linearity Test (Global) |  | < 0.001 |

| **Table S9** Multivariable cox regression analysis association between MAR and **CVD mortality**. | | | | | | | | | | |
| --- | --- | --- | --- | --- | --- | --- | --- | --- | --- | --- |
|  | **HR** | | | | | | | | |  |
| **MARx100** | **NO.** | **n(%)** | **Crude Model** | ***P* Value** | **Model 1** | ***P* Value** | **Model 2** | ***P*-Value** | **Model 3** | **P-Value** |
| Continuous | 2022 | 985  (48.7) | 1.42  (1.19~1.7) | <0.001 | 1.34  (0.95~1.49) | 0.161 | 1.23  (1.0~1.51) | 0.056 | 1.17 (0.94~1.45) | **0.154** |
| **Tertile** |  |  |  |  |  |  |  |  |  |  |
| T1  (< 1.19) | 649 | 209 (32.2) | 1(Ref) |  | 1(Ref) |  | 1(Ref) |  | 1(Ref) |  |
| T2 (1.19-1.62) | 696 | 263  (37.8) | 1.18 (0.89~1.56) | 0.263 | 1.16  (0.87~1.56) | 0.316 | 1.18 (0.88~1.59) | 0.257 | 1.14 (0.85~1.53) | **0.38** |
| T3  ( ≥ 1.62) | 677 | 513 (75.7) | 1.54 (1.17~2.03) | 0.002 | 1.24  (0.93~1.67) | 0.148 | 1.24 (0.92~1.66) | 0.161 | 1.19  (0.89~1.61) | **0.246** |
| P for Trend |  |  |  | 0.002 |  | 0.151 |  | 0.167 |  | **0.252** |
| **Note: MAR values were multiplied by 100 for presentation purposes. HRs represent the effect per 1-unit increase in MAR (×100).** MAR values were converted to multiples of 100 for analysis. Cox regression models were constructed to estimate hazard ratios (HRs) with 95% confidence intervals (CIs). **Model 1** was adjusted for age, sex, race, PIR, education, smoking status, alcohol consumption, and BMI; **Model 2** was adjusted for Model1 + CHF, CHD, Hyperlipidemia, Hypertension, and DM; **Model 3** was adjusted for Model1 + Model2 + BUN, Cr, TC, UA. T1 served as the reference group, and P for trend was calculated across tertiles. MAR, monocyte-to-albumin ratio; Ref, reference.  **Table S10** Multivariable cox regression analysis association between MAR and **cerebrovascular disease mortality**. | | | | | | | | | | |
|  | **HR** | | | | | | | | |  |
| **MARx100** | **NO** | **n(%)** | **Crude Model** | ***P*-Value** | **Model 1** | ***P*-Value** | **Model 2** | ***P*-Value** | **Model 3** | **P-Value** |
| Continuous | 2022 | 985  (48.7) | 1.41 (1.02~1.95) | 0.038 | 1.34 (0.95~1.89) | 0.1 | 1.32 (0.94~1.87) | 0.112 | 1.23 (0.86~1.76) | **0.252** |
| **Tertile** |  |  |  |  |  |  |  |  |  |  |
| T1  (<1.19) | 649 | 209 (32.2) | 1(Ref) |  | 1(Ref) |  | 1(Ref) |  | 1(Ref) |  |
| T2 (1.19-1.62) | 696 | 263  (37.8) | 1.11 (0.68~1.81) | 0.682 | 1.35 (0.81~2.26) | 0.25 | 1.35 (0.80~2.25) | 0.26 | 1.26 (0.75~2.11) | 0.387 |
| T3  (≥1.45) | 677 | 513 (75.7) | 1.4  (0.86~2.27) | 0.176 | 1.44 (0.85~2.45) | 0.171 | 1.4  (0.83~2.37) | 0.205 | 1.28 (0.76~2.18) | **0.352** |
| P for Trend |  |  |  | 0.178 |  | 0.171 |  | 0.206 |  | **0.354** |

**Note: MAR values were multiplied by 100 for presentation purposes. HRs represent the effect per 1-unit increase in MAR (×100).** MAR values were converted to multiples of 100 for analysis. Cox regression models were constructed to estimate hazard ratios (HRs) with 95% confidence intervals (CIs). **Model 1** was adjusted for age, sex, race, PIR, education, smoking status, alcohol consumption, and BMI; **Model 2** was adjusted for Model1 + CHF, CHD, Hyperlipidemia, Hypertension, and DM; **Model 3** was adjusted for Model1 + Model2 + BUN, Cr, TC, UA. T1 served as the reference group, and P for trend was calculated across tertiles. MAR, monocyte-to-albumin ratio; Ref, reference.

| **Table S11** Multivariable cox regression analysis association between inflammatory markers and all-cause mortality | | | | | | | | | | | | | |
| --- | --- | --- | --- | --- | --- | --- | --- | --- | --- | --- | --- | --- | --- |
|  | **HR** | | | | | | | | |  |  |  |  |
| **variable** | **NO.** | **n(%)** | **Crude Model** | ***P*-Value** | **Model 1** | ***P*-Value** | **Model 2** | ***P*-Value** | **Model 3** | ***P*-Value** | **Model 4** | ***P*-value** |  |
| MAR,  100xconversion | 2022 | 985 (48.7) | 1.72  (1.52~1.94) | <0.001 | 1.32  (1.13~1.53) | <0.001 | 1.32  (1.13~1.54) | <0.001 | 1.35  (1.19~1.53) | <0.001 | **1.39**  **(1.22–1.59)** | **<0.001** |  |
| NLR | 2022 | 985 (48.7) | 1.14  (1.11~1.17) | <0.001 | 1.12  (1.09~1.16) | <0.001 | 1.12  (1.08~1.16) | <0.001 | 1.12  (1.08~1.15) | <0.001 |  |  |  |
| PLR, z-score | 2022 | 985 (48.7) | 1.12  (1.05~1.19) | <0.001 | 1.1  (1.02~1.18) | 0.018 | 1.11  (1.03~1.2) | 0.004 | 1.1  (1.03~1.19) | 0.007 |  |  |  |
| SII, z_score | 2022 | 985 (48.7) | 1.15  (1.1~1.2) | <0.001 | 1.12  (1.07~1.17) | <0.001 | 1.1  (1.05~1.15) | <0.001 | 1.08  (1.03~1.13) | 0.001 |  |  |  |
| PNI | 2022 | 985 (48.7) | 0.93  (0.91~0.95) | <0.001 | 0.93  (0.91~0.95) | <0.001 | 0.93  (0.91~0.96) | <0.001 | 0.94  (0.91~0.96) | <0.001 |  |  |  |
| Albumin, g/L | 2022 | 985 (48.7) | 0.93  (0.91~0.95) | <0.001 | 0.93  (0.91~0.95) | <0.001 | 0.93  (0.91~0.96) | <0.001 | 0.94  (0.91~0.96) | <0.001 |  |  |  |
| Monocyte count | 2022 | 985 (48.7) | 2.81  (2.23~3.55) | <0.001 | 1.72  (1.25~2.36) | 0.001 | 1.77  (1.29~2.45) | <0.001 | 1.7  (1.23~2.35) | 0.001 |  |  |  |

**Crude model**: no adjustment was made for any covariate.

**Model 1** was adjusted for age, sex, race, PIR, education, smoking status, alcohol consumption, and BMI;

**Model 2** was adjusted for Model1 + CHF, CHD, Hyperlipidemia, Hypertension, and DM;

**Model 3** was adjusted for Model1 + Model2 + BUN, Cr, TC, and UA.

Notes: The data for MAR is presented as per 100x conversion, PLR and SII were respectively converted using the Z-Score transformation.

Abbrivattion: HR,hazard Ratio;95%CI,95% confidence interval; MAR, monocyte - to - albumin ratio; Ref, reference; PIR, poverty income ratio, Ref: reference; PIR: poverty income ratio; BMI: Body Mass Index; DM: Diabetes Mellitus, CHF, congestive heart failure.

**Figure S4** Comparison of Predictive Accuracy for All-Cause Mortality Among Novel Inflammatory Markers in Stroke Cohort.

Note: The figure displays receiver operating characteristic (ROC) curves for various inflammatory and nutritional biomarkers in predicting long-term mortality. The y-axis represents sensitivity, and the x-axis represents 1-specificity. The area under the curve (AUC) quantifies the discriminatory ability of each marker. Seven biomarkers were compared: Monocyte-to-Albumin Ratio (MAR), Prognostic Nutritional Index (PNI), Systemic Immune-Inflammation Index (SII), Neutrophil-to-Lymphocyte Ratio (NLR), Platelet-to-Lymphocyte Ratio (PLR), Serum Albumin (g/dL), and Monocyte Count (x 10⁹/L).

**Table S12** Incremental Predictive Value of Adding MAR to the Conventional Risk Model for All-Cause Mortality in Stroke Survivors

| **Follow time** | **C-statistic (95%CI)** | | | **Continunous NRI (95%CI)** | **P value** | **IDI (95%CI)** | **P value** | |
| --- | --- | --- | --- | --- | --- | --- | --- | --- |
|  | **BaseModel*** | **BaseModel+MAR** | |  |  |  |  |  |
| All cause mortality | | | | | | | |  |
| 3 year | 0.513  (0.347~0.680) | | 0.590  (0.422~0.758） | -0.019 | 0.144 | -0.019 | 0.754 | |
| 5 year | 0.544  (0.427~0.661) | | 0.605  (0.490~0.720) | 0.219 (0.005~0.412) | 0.04 | 0.002(–0.001~0.005) | 0.14 | |
| 8 year | 0.580  (0.496~0.665) | | 0.678  (0.597~0.760） | 0.258 (0.056~0.395) | 0.004 | 0.005(0.001~0.012) | 0.016 | |
| 10 year | 0.594  (0.523~0.665) | | 0.680  (0.608~0.751） | 0.254 (0.091~0.37) | <0.001 | 0.024(0.007~0.06) | <0.001 | |
| 15 year | 0.600  (0.566~0.681) | | 0.659  (0.603~0.715） | 0.207 (0.095~0.309) | <0.001 | 0.019(0.006~0.045) | <0.001 | |
| 20 year | 0.596  (0.552~0.640) | 0.642  (0.594~0.690） | | 0.176 (0.091~0.267) | <0.001 | 0.018 (0.007~0.038) | <0.001 | |

**Table S13** Multivariate regression analysis between MAR and all-cause mortality for different MAR cutoff values.

| **MAR(x100)** | **HR** | | | | | | | | |  |
| --- | --- | --- | --- | --- | --- | --- | --- | --- | --- | --- |
|  | **NO.** | **n(%)** | **Crude Model** | ***P*-Value** | **Model 1** | ***P*-Value** | **Model 2** | ***P*-Value** | **Model 3** | **P-Value** |
| ****Tertile**** |  |  |  |  |  |  |  |  |  |  |
| T1 ( < 1.19) | 649 | 209 (32.2) | 1(Ref) |  | 1(Ref) |  | 1(Ref) |  | 1(Ref) |  |
| T2 (1.19-1.62) | 696 | 263  (37.8) | 1.16 (0.98~1.36) | 0.079 | 1.12  (0.95~1.32) | 0.194 | 1.11 (0.94~1.31) | 0.203 | 1.05 (0.89~1.24) | 0.551 |
| T3 ( ≥ 1.62) | 677 | 513 (75.7) | 1.75 (1.5~2.04) | <0.001 | 1.40 (1.19~1.65) | <0.001 | 1.39 (1.18~1.63) | <0.001 | 1.32 (1.12~1.55) | 0.001 |
| P for Trend |  |  |  | <0.001 |  | <0.001 |  | <0.001 |  | 0.001 |
| **Youden index** |  |  |  |  |  |  |  |  |  |  |
| < 1.518 | 1188 | 512 (43.1) | 1(Ref) |  | 1(Ref) |  | 1(Ref) |  | 1(Ref) |  |
| ≥ 1.518 | 834 | 473 (56.7) | 1.67 (1.47~1.89) | <0.001 | 1.29 (1.11~1.51) | <0.001 | 1.29 (1.11~1.51) | 0.001 | **1.24** (1.06~1.45) | **0.009** |

Note: MAR values were multiplied by 100 prior to analysis. The table is divided into two sections based on different cutoff strategies. The first section presents the results using **tertile-based cutoffs** (T1: <1.19, T2: 1.19–1.62, T3: ≥1.62), which were utilized to identify independent risk predictors with a dose-response relationship. The second section displays the Cox regression models using the **optimal cutoff value of 1.518**, determined by the Youden index from the Receiver Operating Characteristic (ROC) curve analysis (Figure S5), to categorize patients into High-Risk (≥1.518) and Low-Risk (<1.518) groups for clinical application. HR = Hazard Ratio; CI = Confidence Interval.

**Figure S5** Restricted cubic spline analysis illustrating the dose-response relationship between MAR (log2) and all-cause mortality in the sensitivity analysis using data restricted to the 0.1th–99th percentiles of MAR distribution.

Note: The analysis was performed after excluding extreme outliers (the 0.1th–99th percentiles of MAR distribution) as a sensitivity analysis. P for overall association was <0.001, and Pfor non-linearity was 0.015, indicating a significant deviation from a linear trend. The red line represents the estimated hazard ratio (HR), and the shaded area denotes the 95% confidence interval (CI). A reference point (HR=1) was set at MAR = 0.4806. The histogram illustrates the distribution density of the study population across MAR values.

**Table S14** Sensitivity Analysis Addressing Potential MNAR of Albumin.

| **Variable** | **Crude Model** | ***P*-Value** | **Model 1** | ***P*-Value** | **Model 2** | ***P*-Value** | **Model 3** | **P-Value** |
| --- | --- | --- | --- | --- | --- | --- | --- | --- |
| MAR, (x100) | 1.57 (1.46~1.69) | <0.001 | 1.39 (1.27~1.51) | <0.001 | 1.39 (1.28~1.52) | <0.001 | 1.34 (1.23~1.47) | <0.001 |
| Albumin,g/L | 0.95 (0.94~0.96) | <0.001 | 0.96 (0.94~0.97) | <0.001 | 0.96 (0.94~0.97) | <0.001 | 0.96 (0.95~0.97) | <0.001 |
| Monocyte count, x10^9^/L | 2.86 (2.33~3.51) | <0.001 | 1.97 (1.55~2.49) | <0.001 | 2.01 (1.58~2.55) | <0.001 | 1.81 (1.42~2.31) | <0.001 |

**Crude model**: no adjustment was made for any covariate.

**Model 1** was adjusted for age, sex, race, PIR, education, smoking status, alcohol consumption, and BMI;

**Model 2** was adjusted for Model1 + CHF, CHD, Hyperlipidemia, Hypertension, and DM;

**Model 3** was adjusted for Model1 + Model2 + BUN, Cr, TC, UA.

Note: Missing albumin values were imputed under a hypothetical MNAR scenario using the clinical cutoff of 35 g/L (best-case assumption), representing a situation where all non-respondents had normal nutritional status. This analysis was conducted to evaluate whether the complete-case results (adjusted for age, sex, race, and poverty income ratio) would be materially biased if missingness depended on unobserved low albumin levels.

Abbreviate: OR = odds ratio; CI = confidence interval.

**Table S15** Sensitivity analysis of the incremental predictive value of the MAR when added to a fully adjusted baseline model in stroke survivors..

| **Measure** | **BaseModel*** | **BaseModel+MAR** | **Improvement** | **95%CI** | **P value** |
| --- | --- | --- | --- | --- | --- |
| **AUC / C-statistic** | 0.640 | 0.6512 | 0.0112 | (0.606, 0.654) | 0.035 |
| **IDI** | - | - | 0.0063 | (0.0028, 0.0099) | < 0.001 |
| **NRI (Continuous)** | - | - | 0.1877 | (0.1015, 0.274) | < 0.001 |
| **NRI (Category)** | - | - | 0.0309 | (0.0072, 0.0546) | 0.01 |

**Note: *Base model included age, sex, smoke status, alcohol consumption, hypertension, diabetes mellitus, coronary heart disease. Angina, heart attack, congestive heart failure, BUN, Cr, eGFR. For the continuous NRI, no predefined risk cut-offs were used. For the categorical NRI, risk strata were defined as <10%, 10%–20%, and ≥20% based on clinical relevance. Statistical significance was determined by the DeLong test for AUC comparison and Z-test for IDI/NRI. The categorical NRI indicator demonstrated a statistically significant improvement in risk reclassification (P = 0.01), with an improvement rate of 3.09% (NRI = 0.0309).**

**Abbreviate: DM, diabetes mellitus; CHF, congestive heart failur; BUN, Blood Urea Nitrogen; Cr, Serum Creatinine; eGFR, estimated Glomerular Filtration Rate. AUC, area under the receiver operating characteristic curve; IDI, integrated discrimination improvement; NRI, net reclassification improvement.**

**Figure S6** Composite evaluation of predictive performance for Model 1 and Model 2 in saturated model.

**Note: Model 1 includes smoke status, alcohol consumption, hypertension, diabetes mellitus, coronary heart disease. Angina, heart attack, congestive heart failure, BUN, Cr, eGFR; Model 2 includes Model 1 plus MAR. Panel A is a receiver operating characteristic ((ROC) curve that shows Model 2 has a significantly higher area under the curve (AUC: 0.6512 vs 0.64, p =0.035) than Model 1, indicating superior discrimination between events and non-events. Panel B is a calibration plot that demonstrates better alignment between predicted probabilities and observed outcomes for Model 2 (red line), with its curve closer to the ideal 45° diagonal than that of Model 1 (blue line), reflecting more accurate risk estimation. Panel C is a decision curve analysis (DCA) that reveals Model 2 yields slightly net benefit than Model 1 and the "treat all" strategy across most clinically relevant risk thresholds, confirming enhanced clinical mildly. Panel D is a time-dependent AUC that illustrates Model 2 (red line with shaded 95% confidence interval) consistently outperforms Model 1 (blue line) over 250 months of follow-up, with both models maintaining stable predictive performance.**

**Figure S7**. E‑value bias plot for the association between MAR and all‑cause mortality.

**Note:** This plot illustrates the relationship between the risk ratio for the exposure-confounder association (RREU) and the risk ratio for the confounder-outcome association (RRUD). The blue line represents the calculated overall risk ratio (RR) based on the formula RR=RREU +RRUD −1RREU×RRUD. The red star indicates the specific scenario where both RREU and RRUD are equal to 2.04. The dashed horizontal line likely represents the null value (RR=1), indicating no association.
